# STING expression is an independent prognostic factor in patients with mycosis fungoides

**DOI:** 10.1038/s41598-022-17122-1

**Published:** 2022-07-26

**Authors:** Reiko Takayanagi-Hara, Yu Sawada, Hitomi Sugino, Yoko Minokawa, Hikaru Kawahara-Nanamori, Misa Itamura, Tomoko Tashiro, Ayaka Kaneoka, Natsuko Saito-Sasaki, Kayo Yamamoto, Etsuko Okada

**Affiliations:** Department of Dermatology, University of Occupational and Environmental Health, 1-1, Iseigaoka, Yahatanishi-Ku, Kitakyushu, 807-8555 Japan

**Keywords:** Immunology, Diseases

## Abstract

Mycosis fungoides is recognized as an indolent cutaneous malignant T-cell lymphoma. In contrast, there are few therapeutic options for advanced forms of mycosis fungoides. Since immunotherapy is desirable as an alternative therapeutic option, identifying candidate molecules is an important goal for clinicians. Although tumor-derived negative immunomodulatory molecules, such as PD-1/PD-L1, have been identified in various malignancies, the useful positive immunological drivers of mycosis fungoides are largely unknown. We found that the stimulator of interferon (IFN) genes (STING) was highly upregulated in early-stage mycosis fungoides. Immunohistochemical examination revealed different STING staining patterns in patients with mycosis fungoides. Although there were no significant differences in clinical factors’ characteristics, STING expression was associated with the survival of patients with mycosis fungoides. The survival rate was significantly poor in patients with low STING-expressing mycosis fungoides. Univariate and multivariate analyses revealed that low STING expression was associated with an increased hazard ratio. Our results indicate that STING expression independently influences the prognosis of mycosis fungoides.

## Introduction

Mycosis fungoides (MF) is the most common subtype of malignant cutaneous T-cell lymphoma, exhibiting epidermotrophic infiltration properties^1^. This malignant tumor shows indolent clinical behavior, and the early-phase is clinically observed as macules and plaques^1,2^. However, this tumor gradually develops into an advanced form of MF and shows a poor clinical course with extracutaneous involvement (lymph nodes, blood or, less commonly, other organs) or large cell transformation^3^. Advanced cases do not ever achieve satisfactory clinical outcomes through current therapeutic options. Therefore, additional candidate therapeutic molecules must be identified.

The presence of anti-tumor immunity has been reported in various malignant tumors; thus, it is imperative for clinicians to overcome the limitations of current systemic chemotherapy against cancers. Indeed, immune checkpoint inhibitors, such as anti-PD-1 antibody treatment, showed a dramatic improvement in clinical outcomes in advanced stages of cancers^4,5^. As a representative tumor, malignant melanoma is an intractable malignancy for which favorable clinical outcomes through treatment with immune checkpoint inhibitors are possible^6^. Because spontaneous regression of the tumor has been recognized in malignant melanoma, the importance of an anti-tumor immune response has been suggested^7,8^. Similarly, the importance of immunological responses in cutaneous lymphomas has also been reported. Understanding spontaneous repression may aid our understanding of the anti-tumor immunological mechanisms against lymphomas^9–14^. Furthermore, previous studies have identified the therapeutic potential of immunotherapy for cutaneous lymphomas. Interferon (IFN) immunotherapy treatment, for instance, shows therapeutic efficacy against lymphoma^15^. In addition, immune checkpoint inhibitor treatment has shown better therapeutic efficacy against lymphoma^16^. These findings demonstrate the therapeutic potential of an anti-tumor immune response against lymphoma. In contrast, some of lymphoma cases showed worsened by this treatment^17^ and only a few studies exist regarding the effects of immunotherapy against lymphomas.

Among the various immunological response drivers, the stimulator of interferon (IFN) genes (STING) was identified as an endoplasmic reticulum adaptor that facilitates innate immune signaling. STING is a protein responsible for controlling anticancer immune responses to self- or non-self-DNA released from dead cells^18,19^. STING has recently been highlighted as a strong type I IFN driver that exhibits antitumor immunity against various malignancies^20^. However, the actual effect of STING on cutaneous lymphomas remains unclear.

In this study, we found that STING was highly expressed in MF and investigated the differential expression of STING in the tumor cells of patients with MF. We identified the prognostic importance of STING expression in MF using statistical analysis.

## Results

### TMEM173 (STING) was the most expressed immune positive driver in early-stage MF

**Figure 1:**
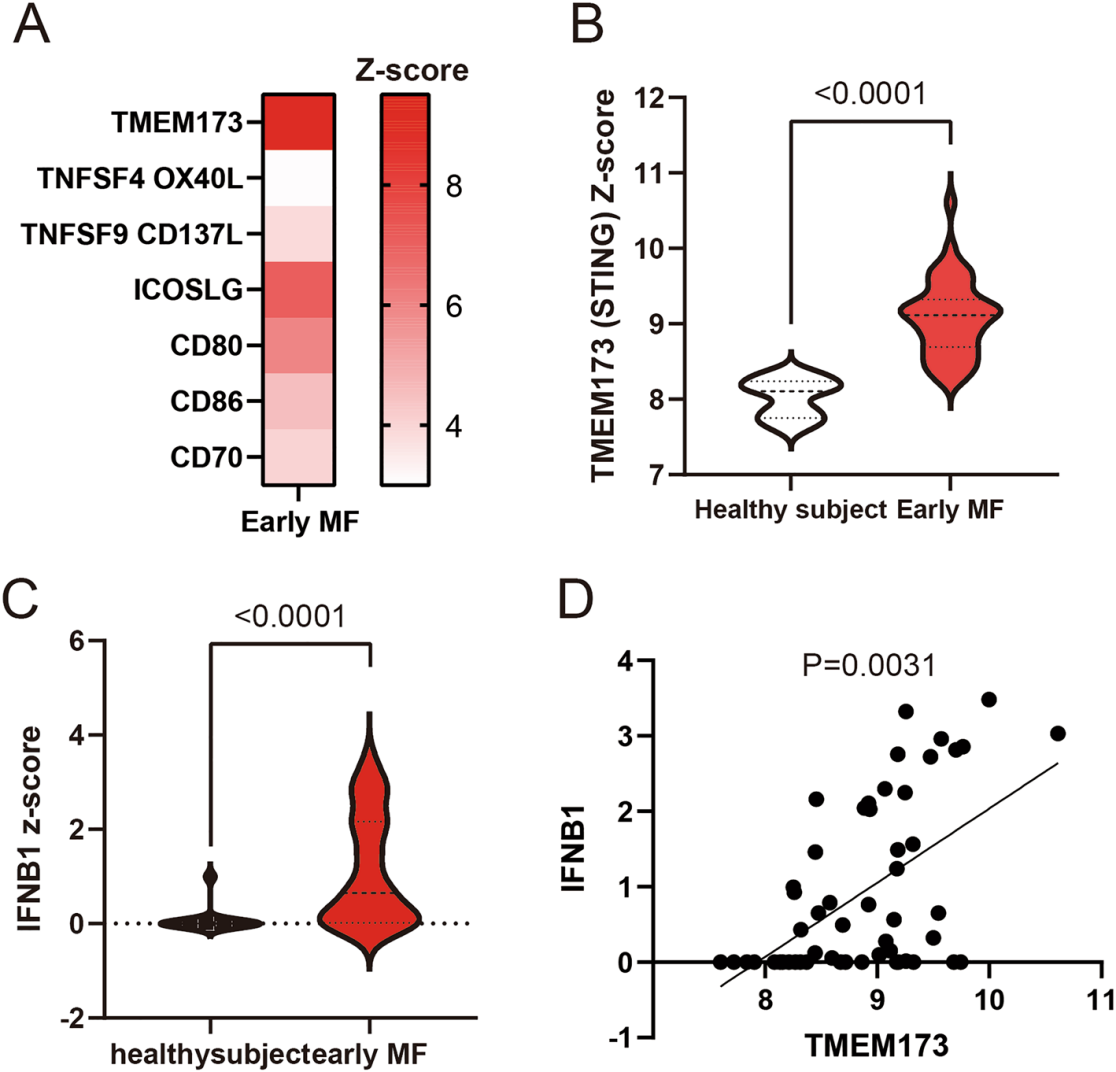
RNA sequencing analysis. (**A**) The different gene expression of positive immune drivers by Heat-map analysis. (**B**, **C**) The gene expressions. The difference of gene expression of TMEM173 (**B**) and IFNB1 (**C**). (**D**) The correlation of the gene expression of TMEM173 and IFNB1.

Immunological positive drivers for the induction of anti-tumor immunity against MF are one of the most relevant factors. Because the natural immunological response to the tumor might not be detected under in vitro-established tumor microenvironments, we first investigated the immunological phenotypes of tumors in patients with early-stage MF, looking for possible therapeutic candidates for MF to reveal the early phase characteristics of the tumor. Recent studies identified several candidates of positive immune drivers in the tumor environment, and RNA-sequencing analysis revealed that TMEM173 was the most upregulated among representative immune-positive drivers in early-stage MF (Fig. 1A). Consistently, gene expression was significantly increased in early-stage MF compared to that in healthy individuals (Fig. 1B). TMEM173 is the STING-encoding gene, and STING has recently been identified as an immunologically positive driver for anti-tumor immune response induction. Therefore, we speculated that STING may play a role in MF.

STING enhances type I IFN production mediated by intracellular pathogens, such as bacteria and viruses, and is essential as a positive immune driver^21^. DNA in the cytoplasm is brought by intracellular viruses or released from the nucleus and mitochondria^22^. It is then recognized by a DNA sensor protein known as cGAS, which activates 2′3’-cyclic GMP-AMP (2′3’-cGAMP) synthase. cGAMP acts as a secondary messenger for STING activation^21^. Type I IFN enhances antigen presentation and T cell proliferation. Therefore, the STING-mediated signaling pathway is desired to enhance anti-tumor immunity against cutaneous malignancies. To explore the importance of STING expression in MF, we also examined IFN-β gene (IFNB1) expression in MF by RNA-sequencing analysis and found that IFNB1 was significantly increased in early-stage MF (Fig. 1C). In addition, STING expression showed a significant positive correlation with IFNB1 expression (Fig. 1D). These findings suggest that STING expression may influence MF prognosis.

### The different clinical characteristics of STING expression in MF

**Figure 2:**
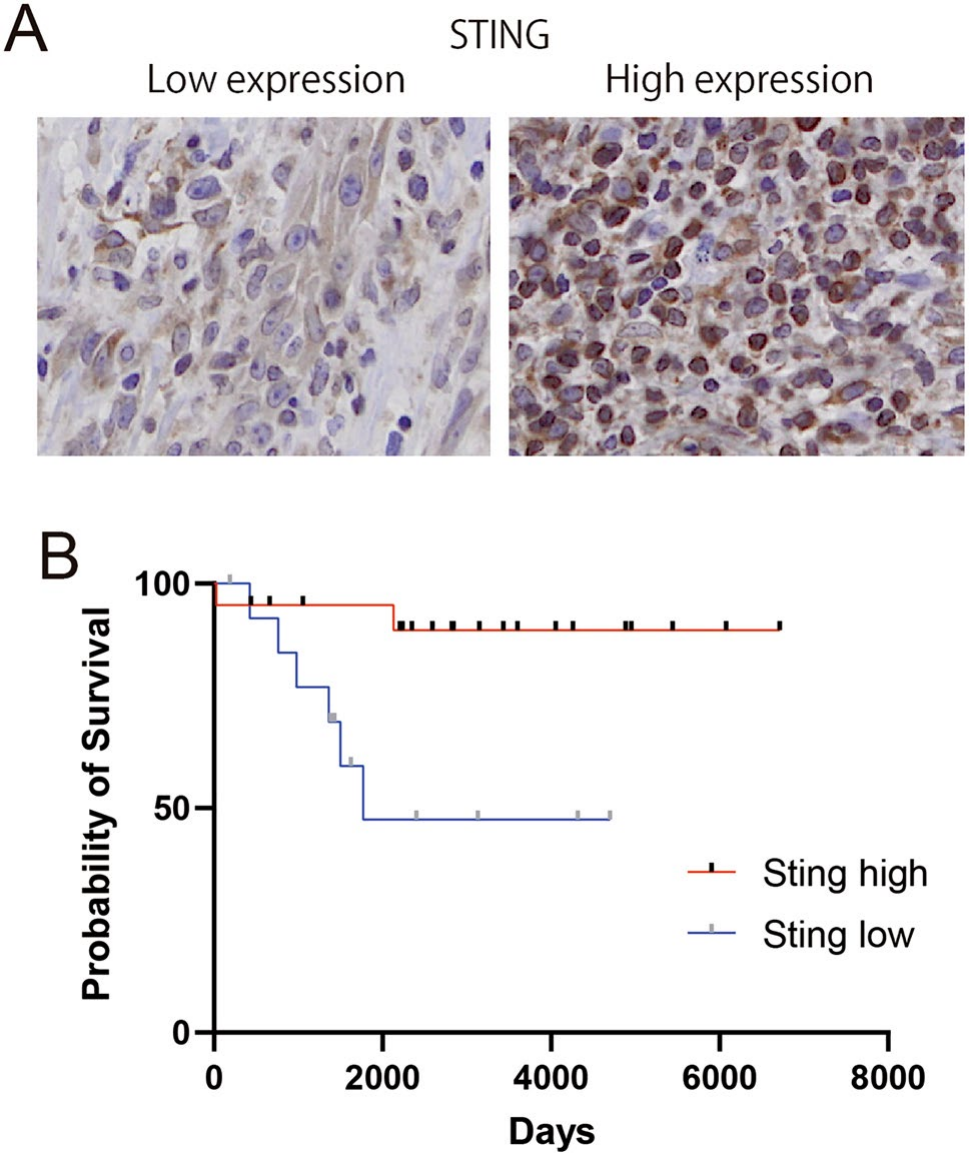
Immunostaining and the survival curve. (**A**) The representative different STING expression in the tumor. Immunostaining for STING in the tumor of mycosis fungoides patients was conducted. (**B**) The survival curves. The difference of survival was calculated by Kaplan–Meier analyses.

We next explored the distribution of STING expression in MF tumor cells. Immunohistochemical staining for STING was performed to reveal its expression pattern. STING was mainly expressed in the cytoplasm of the tumor cells (Fig. 2B). We also noticed two cell populations with different STING expression patterns in the tumor: high- and low-STING expression. Moreover, STING expression was low in tumors with nuclear enlargement (Fig. 2A). Therefore, we speculated that these two populations may show different clinical characteristics of MF. To investigate the differences in clinical characteristics between these two populations, we categorized the clinical characteristics according to the difference in STING expression, as shown in Table 1. However, there were significant differences in these clinical variables between the high- and low-STING expression groups. Therefore, we hypothesized that there was a direct prognostic impact of tumor STING expression in patients with MF.

**Table 1:** The difference of clinical characteristics in STING expression.

Variables	STING expression		
	High	Low	*P*-value
Total number	21	14	–
**Age (70 > or 70 <)**			0.2882
70 >	15	7	–
70 <	6	7	–
**Sex**			
Male	14	10	> 0.9999
Female	7	4	–
**WBC (7000 > or 7000 <)**			0.4875
7000 >	9	4	–
7000 <	12	10	–
**Stage**			0.4830
Early-stage	14	7	–
Late-stage	7	7	–

### STING expression was associated with a higher survival of MF

Previous studies have shown that STING can drive antitumor immune responses and is progressively becoming of interest as a therapeutic target for various tumors. STING also has a direct effect on tumor cell apoptosis. However, the therapeutic potential of STING in cutaneous lymphomas remains unclear. To clarify the prognostic influence of STING in MF, we investigated the difference in survival between high- and low-STING expression in patients with MF. The effects of STING expression on the prognosis of patients with MF were analyzed using the Kaplan–Meier method. The survival rate was significantly poorer in patients with low STING expression compared to patients with high STING expression (Fig. 2B), suggesting that STING expression influences the survival of patients with MF.

### STING expression was an independent prognostic factor in MF

Various clinical characteristics influence the prognosis of MF. Our study identified that the degree of STING expression in MF increased in patients with a better prognosis for MF. Therefore, it is necessary to explore the importance of STING expression as an independent clinical factor in MF, excluding the influence of other variables. Univariate analysis showed that low STING expression significantly increased the hazard ratio in patients with MF (Table 2). We also performed a multivariate analysis to exclude the influence of other clinical variables on the impact of prognosis in our study population. Among these clinical factors, low STING expression was associated with an increased hazard ratio (Table 2). These findings indicate that STING expression independently influences the prognosis of MF.

**Table 2:** Univariate and multivariate analysis.

Variables	Univariate		Multivariate	
	HR	*P*-value	HR	*P*-value
Age	1.294 (0.647–2.589)	0.466	3.350 (0.410–27.405)	0.260
Sex	1.152 (0.562–2.362)	0.700	1.045 (0.228–4.789)	0.954
WBC	1.183 (0.532–2.635)	0.680	1.264 (0.233–6.858)	0.786
Stage	0.762 (0.181–3.210)	0.711	0.535 (0.060–4.758)	0.575
STING expression	5.351 (1.061–26.999)	0.042	12.691 (1.416–113.739)	0.023

## Discussion

In this study, we first showed that the different patterns of STING expression in MF tumor cells play a role in the condition’s prognosis, with a higher expression of STING promoting favorable clinical outcomes. We also showed that STING expression is an independent factor for predicting the prognosis of patients with MF.

STING plays a crucial role in anti-tumor immune responses in various tumors^20^. STING drives downstream type I IFN production, which enhances the anti-tumor immune response. Our findings indicate that STING expression is positively correlated with IFNB1 expression. Although our study could not confirm the direct action of STING on MF, tumor-expressed STING is expected to enhance the anti-tumor immune response against MF, and is closely related to survival.

STING is expected to drive an anti-tumor immune response mediated by downstream cytokines such as IFN-β. IFN-β activates dendritic cells, leading to T cell proliferation^23^. These cells are expected to elicit anti-tumor immune responses against malignancies. In contrast, IFN-β treatment has shown unsatisfactory therapeutic outcomes in cutaneous lymphomas. Therefore, STING-mediated therapeutic benefits seem not to be only STING-IFN-mediated, but other underlying therapeutic effects might exist. One possibility is that STING becomes a trigger to enhance the DNA damage response in tumor cells^24^. Furthermore, STING can enhance major histocompatibility complex (MHC) class I expression in tumors and immune cells^25^. Therefore, these additional therapeutic effects of STING might also be involved in the development of STING-mediated antitumor effects.

Although a radical therapeutic option for the advanced form of MF is available, immune checkpoint inhibitor treatment is also an option and shows favorable clinical outcomes. Therefore, STING-targeted anti-tumor immunotherapy may be a therapeutic candidate for future treatment^19^. Our study showed that a possible potent STING signaling drives an anti-tumor immune response against MF, suggesting that a therapeutic tool able to promote STING-bearing tumors is another therapeutic candidate for MF treatment. The current immune checkpoint inhibitor treatment might also enhance therapeutic outcomes in combination with STING-targeted therapy.

Several clinical studies have investigated the prognostic significance of STING expression in various malignancies. However, these results are controversial and vary depending on the type of malignancy. A clinical study showed that lower expression of STING in gastric cancer patients was associated with poorer survival in addition to clinical stage and tumor size progression^26^. Furthermore, multivariate analysis identified STING as an independent prognostic factor of overall survival^26^. In contrast, another recent study showed that in EB virus-positive gastric cancer, STING-expressing cancer cells had better prognosis, while in EB virus-negative gastric cancer STING-expressing cancer cells had poorer prognosis^27^. In lung cancers, non-small cell lung cancer showed a better prognosis in tumors with lower STING expression, and STING-expressing tumors showed a significantly higher frequency of EGFR and KRAS mutations^28^. In contrast, STING expression was not associated with tumor size, clinical stage, or survival in colorectal cancer^29^. These findings suggest that the prognostic influence of STING may depend on the type of malignancy; therefore, it is necessary to investigate the detailed influences of STING in individual cancers.

Because STING expression is significantly related to favorable clinical behavior, STING expression may be a useful biomarker for predicting the prognosis of MF. Consistent with our results, the existence of the STING signaling activation pathway might be a reasonable explanation for the infiltration of lymphocytes around MF tumor cells.

As a limitation of our study, we could not evaluate the blood involvement of the tumor by flow cytometry. Recent update studies revealed the utility of tumor involvement in the blood^30^, while flow cytometry analysis is clinically often performed only in patients with the advanced-stage disease^31,32^. And some recommendations for cases where the use of flow cytometry in patients with MF is especially some appropriate conditions, such as a patient with advanced-stage disease (stage IIB and above), or lack of response to treatment^32^. Therefore, we could not completely deny the possibility of underestimation of the clinical stage based on the evaluation of blood involvement by manual counting of atypical cells in peripheral blood.

Taken together, the prognosis of MF depends on the degree of STING expression, which can be used to predict survival mediated by the anti-tumor immune response to MF. STING signaling is a strong anti-tumor immune driver and is expected to overcome the limitations of the current therapeutic efficacy against MF.

## Methods

### Patients

Thirty-five patients with MF were enrolled in this study. MF was diagnosed based on histological and clinical examinations. These patients were seen at the University of Occupational and Environmental Health from April 2000 to March 2021. The clinical factors evaluated were age, sex, WBC count, presence of atypical lymphocytes, and clinical stage. We defined and classified stage IA-IIA as early-stage MF and stages IIB-IV as late-stage MF based on the findings at the first visit to our department^33^.

### Histology and immunohistochemistry

Immunohistochemical staining for STING was conducted as previously described, though with several modifications^34^. In brief, section specimens were deparaffinized and hydrated by xylene washing and treated with a graded series of alcohol. For treatment with unmasking antigens, section specimens were treated with 10 mM citric acid (pH 6) (Dako) at 95 °C for 30 min and then blocked with normal sera at room temperature for 60 min. The specimens were then incubated with a primary antibody (STING; Proteintech, Wuhan, China). After washing the samples, the sections were incubated at room temperature for 30 min with secondary antibodies, and the targeted antigens were visualized by 3,3-diaminobenzidine staining. To evaluate the degree of STING-positive cells in the sections precisely, the frequencies of immunoreactive lymphocyte were counted in each immunostaining section. Tumors containing more than 40% of STING-expressing cells were classified as the high STING-expression group, while those containing less than 40% of STING-expressing cells were classified as the low STING-expression group.

### Statistical analysis

Fisher’s exact test was used to analyze the association between STING staining and clinical parameters. Overall MF survival was analyzed from the date of the first diagnosis in our department to the date of the latest contact with the patient or death. Fisher’s test and Kaplan–Meier survival analyses were conducted using version 9.2.0 GraphPad Prism. The sensor placed on the survival curve indicates discontinuation of follow-up observation or survival during the study period. Univariate and multivariate analyses were conducted using SPSS version 27 (IBM Corp., Armonk, NY, USA).

### Microarray data analysis

Microarray dataset analysis was conducted as previously described^35^. Gene expression in 43 cases of early-stage MF and 12 healthy individuals were investigated using public datasets obtained from the National Center for Biotechnology Information (NCBI) Gene Expression Omnibus (GEO) database (GEO accession no. GSE143382). Gene expression is indicated by a Z-value index. The Student’s t-test was conducted to calculate *P*-values of the differences in gene expression between early-stage MF and healthy individuals.

### Study approval

The University of Occupational and Environmental Health Review Board approved this study, which was performed in accordance with the guidelines of the Declaration of Helsinki. Because this was a retrospective cohort study, the opt-out method was adopted to obtain a waiver of informed consent approved by the ethics committee’s review board of the University of Occupational and Environmental Health.

## Data Availability

The datasets used and/or analysed during the current study available from the corresponding author on reasonable request.
